# Episiotomy in Operative Vaginal Delivery Reduces the Risk of Obstetric Anal Sphincter Injuries in Nulliparous Women: A Systematic Review and Meta-Analysis

**DOI:** 10.3390/jcm15134962

**Published:** 2026-06-25

**Authors:** Andrea Braga, Maurizio Serati, Alessandro Ferdinando Ruffolo, Giorgio Treglia, Giorgio Caccia, Vita Zacesta, Giorgia Tenani, Marco Torella, Matteo Frigerio, Marco Soligo, Stefano Salvatore, Andrea Papadia, Greta Codoni

**Affiliations:** 1 Department of Obstetrics and Gynaecology, EOC—Beata Vergine Hospital, 6850 Mendrisio, Switzerland; giorgio.caccia@eoc.ch (G.C.); vita.zacesta@eoc.ch (V.Z.); giorgia.tenani@eoc.ch (G.T.); greta.codoni@eoc.ch (G.C.); 2 Faculty of Biology and Medicine, Università della Svizzera Italiana, 6900 Lugano, Switzerland; giorgio.treglia@eoc.ch (G.T.); andrea.papadia@eoc.ch (A.P.); 3 Department of Obstetrics and Gynaecology, Del Ponte Hospital, University of Insubria, 21100 Varese, Italy; mauserati@hotmail.com; 4 CHU Lille, Department of Gynaecology, Jeanne de Flandre University Hospital, 59000 Lille, France; alesruffolo@gmail.com; 5 Medical Education and Research Area, General Directorate, Ente Ospedaliero Cantonale, 6500 Bellinzona, Switzerland; 6 Faculty of Biology and Medicine, University of Lausanne, 1011 Lausanne, Switzerland; 7 Department of Gynaecology, Obstetric and Reproductive Science, Second University of Naples, 80138 Naples, Italy; marco.torella@unina2.it; 8 Department of Gynaecology and Obstetrics, San Gerardo Hospital, 20900 Monza, Italy; frigerio86@gmail.com; 9 Department of Obstetrics and Gynaecology, Hospital of Lodi, 26900 Lodi, Italy; dr@marcosoligo.it; 10 Department of Obstetrics and Gynaecology, IRCSS San Raffaele Scientific Institute, 20132 Milan, Italy; 11 Department of Obstetrics and Gynaecology, EOC—Lugano Regional Hospital, 6900 Lugano, Switzerland

**Keywords:** episiotomy, operative vaginal delivery, obstetric anal sphincter injuries, OASIS, vacuum delivery, forceps delivery, severe perineal trauma

## Abstract

**Background:** Although routine use of episiotomy is widely discouraged in spontaneous vaginal deliveries, the use of episiotomy in operative vaginal delivery (OVD) remains highly debated. This systematic review and meta-analysis evaluated the current evidence on the safety of episiotomy in preventing obstetric anal sphincter injuries (OASIS) during OVD. **Methods:** A systematic literature search was conducted on PubMed/MEDLINE and CENTRAL databases with a cut-off date of November 2025. The protocol was registered in PROSPERO. We included studies which encompassed the incidence of OASIS when episiotomy (median or medio-lateral) was performed during vacuum or forceps delivery and when episiotomy was not performed. Randomized controlled trials (RCTs), prospective or retrospective cohort studies and observational studies were considered appropriate study designs. A meta-analysis using risk ratio as the outcome measure was performed to compare the presence of the event (OASIS) in patients with or without episiotomy. **Results:** A total of 15 studies were analyzed to evaluate the role of medio-lateral episiotomy (MLE) in OVD. The results demonstrated a statistically significant lower rate of severe perineal trauma in the experimental group (pooled RR 0.5; 95% CI: 0.30–0.84). A statistically significant difference in support of the use of MLE was found in two further subgroup analyses, comprising 14 studies focusing on the role of MLE during vacuum delivery (VD) and 9 studies considering the role of MLE during forceps delivery (FD). **Conclusions:** This study highlights that the use of MLE in nulliparous women undergoing OVD is associated with a significantly decreased risk of OASIS.

## 1. Introduction

### 1.1. Rationale

Episiotomy, a surgical incision made in the perineum during childbirth, is widely debated in obstetrics. Initially introduced to facilitate delivery, reduce the risk of severe perineal tears and prevent long-term pelvic floor complications, episiotomy became routine in many clinical settings by the 1980s [1]. Since the 1980s, both pregnant women and physicians have begun to question the real benefits of routine episiotomy. This growing skepticism led to the publication of several articles opposing the routine use of the procedure, to the extent of defining it as genital mutilation in certain circumstances [2], culminating in the publication by the World Health Organization (WHO) Technical Working Group of “Care in normal Birth: a practical guide” [3] which recommended a 10% episiotomy rate. However, it was only after the first Cochrane review [4] that a substantial change occurred. The authors claimed that the procedure could lead to complications such as pain, infection, increased blood loss and sexual dysfunction without consistently delivering the expected benefits. Ten years later, the same authors published an updated review advocating a more restrictive use of episiotomy. Their findings showed that a more restrictive approach resulted in less severe perineal trauma, less need for sutures, less posterior perineal injury and fewer healing complications [5]. Moving to the present, the WHO and various national guidelines recommend a limited use of episiotomy, reserving it for specific clinical scenarios rather than applying it routinely [6]. Research comparing routine and restrictive episiotomy consistently reveals that for women expected to have an unassisted vaginal delivery, a selective episiotomy approach may reduce the incidence of severe perineal or vaginal trauma by 30%. Concerning other outcomes, such as perineal infections, perineal pain, bleeding during delivery, long-term urinary incontinence and dyspareunia, genital prolapse, Apgar score and other medium- to long-term effects on the mother or baby, the authors did not identify any statistically significant differences [7]. In addition, this analysis did not rule out midline episiotomy (ME) and the role of episiotomy during OVD was not taken into consideration. As a main conclusion, the latest Cochrane review shows that believing routine episiotomy will reduce this kind of trauma is not supported by the current evidence [7]. While its routine use has been widely discouraged in spontaneous vaginal births, the application of episiotomy in OVD, such as those assisted by forceps or vacuum extraction, remains the subject of ongoing debate. In some studies, a lower incidence of perineal tears has been observed with the use of episiotomy [8,9]. Nevertheless, the evidence supporting the routine use of episiotomy in OVD is not conclusive.

### 1.2. Objective

This updated systematic review and meta-analysis aims to assess the current evidence on the safety of episiotomy use in terms of preventing OASIS during OVD. As clinical guidelines evolve to prioritize patient-centered care, it is crucial to determine whether routine episiotomy during operative deliveries is truly beneficial or if it should be restricted to specific clinical circumstances. Addressing this knowledge gap is essential to refine clinical guidelines and ensure that episiotomy is used appropriately to optimize maternal and neonatal health outcomes.

## 2. Materials and Methods

This study followed the 2020 Preferred Reporting Items for Systematic Reviews and Meta-Analyses (PRISMA) guidelines [10]. The protocol was registered with PROSPERO 2025 CRD420251155634. It is available from https://www.crd.york.ac.uk/PROSPERO/view/CRD420251155634 (accessed on 25 september 2025).

### 2.1. Eligibility Criteria

Included in our analysis were studies which encompassed the incidence of pelvic floor tears in cases where an episiotomy (ME or MLE) was performed during OVD (VD or FD) and in cases where no episiotomy was performed. RCTs, prospective or retrospective cohort studies and observational studies were considered appropriate epidemiological study designs. Review articles, case reports, commentaries, editorials, and meeting abstracts on the selected topic were excluded. Only studies published in the last 30 years were included.

### 2.2. Information Source

The systematic literature search was conducted on PubMed/MEDLINE (Medical Literature Analysis and Retrieval System Online) and CENTRAL (Cochrane Central Register of Controlled Trials) databases with a cut-off date of November 2025.

### 2.3. Search Strategy

The terms used for the literature search, either alone or in combination, were as follows:

- “Episiotomy” AND
- “Operative vaginal delivery” OR “Instrumental delivery” OR “Forceps delivery” OR “Vacuum delivery” AND
- “Obstetric anal sphincter injuries” OR “Severe pelvic floor tears

All relevant articles were thoroughly assessed and their reference lists reviewed to identify additional manuscripts for inclusion in this systematic review and meta-analysis.

### 2.4. Selection Process

Two independent reviewers (A.B. and G.C.) screened each article for title and abstract, excluding studies that were not relevant. Disagreements were resolved by consensus including a third author (M.Se.). Potentially eligible studies were then reviewed in full text and a decision was made on whether to include them in the qualitative and quantitative analyses. Finally, narrative synthesis and quantitative analysis (meta-analysis) were performed.

### 2.5. Data Collection

Using two structured tables, the necessary data was extracted from each included study. The data extracted and then analysed are: authors’ names, year of publication, study design, country, mean age, parity, body mass index (BMI), study aim, outcome definition, type of operative delivery, type of episiotomy, rate of episiotomy, rate of OASIS, results, persistence pain, urinary and anal incontinence. Two independent reviewers (A.B. and G.C.) independently performed the data extraction and collection.

### 2.6. Data Items

- The OASIS rate during OVD with the use of episiotomy (ME and/or MLE).
- The OASIS rate during OVD without the use of episiotomy.
- The rate of Pelvic floor dysfunctions (PFDs), such as persistent pain, urinary or anal incontinence, experienced by women in the post-partum period after OVD with and without episiotomy.

### 2.7. Study Risk of Bias Assessment

The methodological quality of the cohort studies included in this systematic review and meta-analysis was assessed independently by two authors (G.C. and A.B.) using the Newcastle–Ottawa Scale (NOS). In accordance with the established criteria, studies classified as seven to nine stars were deemed to be at low risk of bias (RoB). Those categorized as five to six stars were considered to be at moderate RoB, while those assigned fewer than five stars were classified as high RoB. Any uncertainties were resolved through discussion with a third author (M.Se.).

### 2.8. Statistical Analysis

Continuous variables were expressed as both absolute and relative (percentage) values. A meta-analysis using risk ratio (RR) as the outcome measure was performed comparing the presence of the event (OASIS) in patients with or without episiotomy. Forest plots were used to graphically display the estimated results. Statistical pooling of data was performed using a random-effects model [11]. The results were reported as a pooled RR with associated 95% confidence interval (95–CI) values. The heterogeneity between studies was assessed using the Higgins I2 index [12], with I2 values >50% indicating the presence of heterogeneity [12,13]. Egger’s test and funnel plots were used to assess publication bias [14]. In this context, a *p*-value < 0.05 was deemed to be statistically significant. Subgroup analyses were conducted in instances where significant heterogeneity was identified among the included studies. The statistical analysis was conducted using http://metaanalysisonline.com, a web-based open access tool for the rapid meta-analysis of clinical and epidemiological studies [15].

## 3. Results

### 3.1. Study Selection

**Figure 1:**
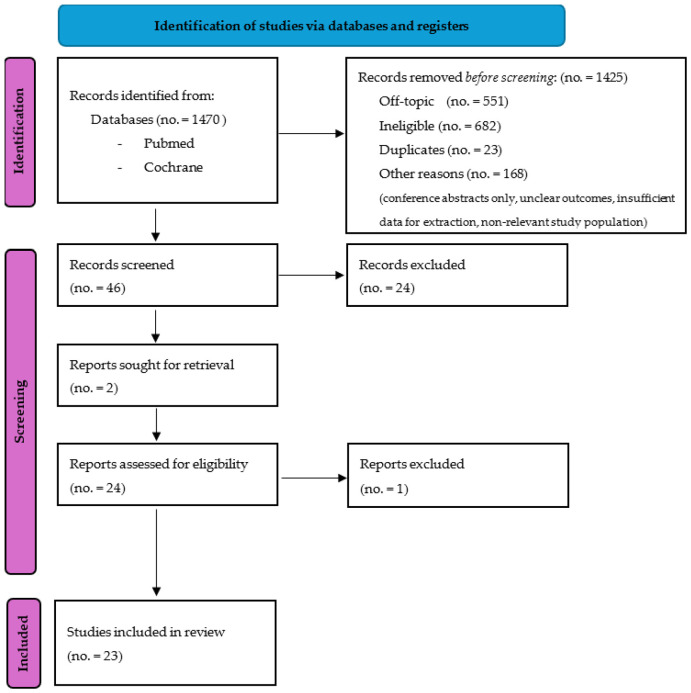
Flow diagram of evidence acquisition in the systematic review and metanalysis.

The comprehensive literature search yielded a total of 1470 articles. After an initial screening based on titles and abstracts, 46 publications were retained for full-text evaluation. Articles were excluded for the following reasons: 551 were not directly related to the research topic, 682 did not meet the eligibility criteria, 23 were duplicates, and 168 were excluded for other reasons. Following this preliminary assessment, 45 articles underwent a second, more detailed review. Of these, 23 studies met the predefined inclusion criteria and were included in the qualitative synthesis, while 17 studies were selected for quantitative analysis. The detailed screening process is illustrated in Figure 1.

### 3.2. Study Characteristics

A total of 23 studies were included in this systematic review and meta-analysis, involving a combined population of 2,360,668 women who underwent OVD, defined as delivery using forceps or vacuum extraction. The characteristics and study designs of the included studies are summarized in Table 1 and Table 2. The studies span a publication period from 1997 to 2023. Among the 23 studies, 18 employed a retrospective design [8,16,17,18,19,20,21,22,23,24,25,26,27,28,29,30,31,32], 3 were prospective studies [32,33,34], and 2 were a pilot randomized controlled trials [35,36]. Ten studies were conducted in a single-center setting [8,16,17,18,23,25,26,29,30,31], whereas 13 were multicenter studies [19,20,21,22,24,27,28,32,33,34,35,36,37]. The included studies originated from 11 different countries, namely the United States, Scotland, the Netherlands, the United Kingdom, Finland, France, Spain, Canada, Israel, Italy and Sweden, covering three continents. Maternal age proved challenging to analyze due to heterogeneity in its reporting across studies, limiting direct comparability. In four studies, age was not specified at all [16,17,18,19], while one study provided only the overall mean age of the included population [26]. Two studies stratified participants into two groups based on age above or below 35 years [33,35], whereas three studies categorized participants by age brackets of 5 [20,27] or 10 years [30].

In ten studies, the mean age was reported separately for subgroups based on whether an episiotomy was performed [8,21,23,24,28,29,31,34,36,37]. Among these, five studies [21,23,24,29,31] found a statistically significant association between age and the likelihood of undergoing episiotomy. Two studies examined age differences in women who sustained an OASIS compared with those who did not [22,25]; of these, one [22] further stratified age by parity without reporting statistical significance, while the other reported age as a significant predictor of OASIS occurrence. Finally, one study [32] investigated the relationship between age and episiotomy angle and concluded that the difference was not statistically significant. Parity was inconsistently reported across the included studies. Ten studies focused exclusively on primiparous women [8,17,23,29,31,32,33,35,36,37], while three studies included both primiparous and multiparous women but did not specify their distribution [16,25,28]. Thirteen studies [18,19,20,21,22,24,25,26,27,30,34] clearly differentiated between primiparous and multiparous participants. After excluding studies that did not report parity-specific data, the pooled population comprised 1,114,745 primiparous and 1,170,724 multiparous women. This allowed for meaningful subgroup analyses based on parity in the majority of the included data. However, a discrepancy of 19,981 women was noted relative to the total study population due to incomplete parity data in several studies [21,22,24,26,27,34], for which this information could not be retrieved for a subset of participants.

BMI was analyzed with considerable heterogeneity across the included studies. Ten studies did not consider BMI as a variable [16,17,18,19,20,21,24,25,27,28]. Three studies reported the proportion of women with a BMI >30 kg/m^2^ [30,33,35], with an average of 13.63% of the study populations falling into this category. Two studies provided only the mean BMI values without further stratification [26,32]. Seven studies [8,23,29,31,34,36,37] examined BMI in relation to the occurrence of episiotomy, with inconsistent findings: some reported a statistically significant association, while others did not. One study [22] investigated BMI in relation to parity and the occurrence of OASIS, but it found no statistically significant associations.

Regarding the type of OVD, five studies [22,29,30,34,36] included only VD. Five studies [17,20,23,27,35] considered both OVD (VD or FD) and vaginal deliveries. In one study, an OVD technique using a spatula was also employed [30].

Most studies reported using the MLE technique. However, in several studies [16,17,18,19,23,28], a percentage of ME was still observed, particularly in older studies, where MLE had not yet become the gold standard. In more recent publications, it is explicitly stated that all cases involved MLE [8,20,21,22,24,25,26,27,29,30,31,32,34,36].

Regarding the persistence of long-term pain after delivery, this aspect was examined in only two studies [33,35]. One study [33] reported that women who underwent episiotomy were more likely to use analgesic medications beyond the first 10 days postpartum compared to those who did not have an episiotomy. The other study [35] focused on the need for analgesia following VD and FD, observing that FD were associated with more prolonged pain compared to VD. Long-term urinary incontinence was examined in three studies [17,33,35], although no significant conclusions were drawn. The same applies to the analysis of long-term anal incontinence.

### 3.3. Risk of Bias in Studies

**Figure 2:**
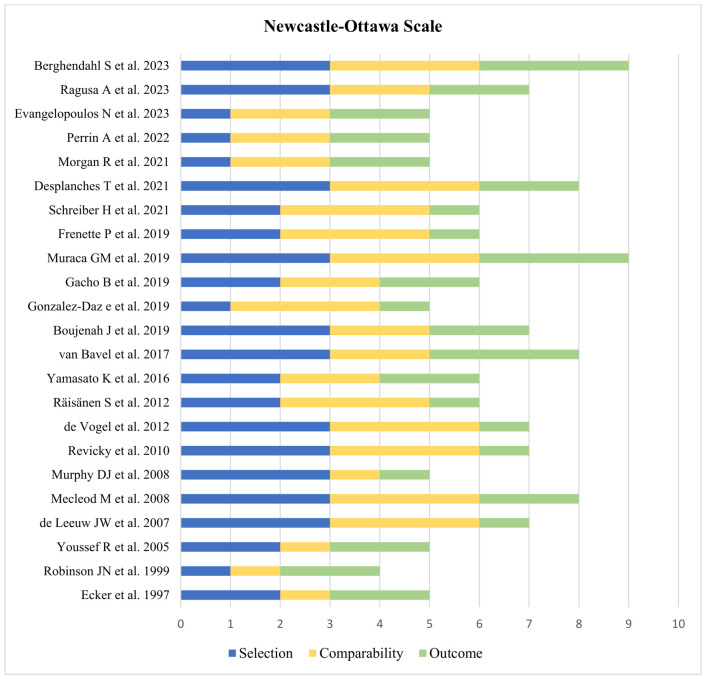
Detailed Newcastle–Ottawa Scale [38] of each included cohort study. We scored the studies across three categories: studies with 7–9 stars were considered of low Risk of Bias (RoB) and studies with 5–6 stars of moderate RoB, whilst studies with less than 5 stars were considered of high RoB [8,16,17,18,19,20,21,22,23,24,25,26,27,28,29,30,31,32,33,34,35,36,37].

As shown in Figure 2, nine studies demonstrated a low RoB, with seven scoring 7/10 [8,19,20,21,22,27,34], four scoring 8/10 [24,29,33,37], and one scoring 9/10 [36] on the NOS. Twelve studies showed a moderate RoB, including seven with a score of 5/10 [16,18,25,30,31,32,35] and four with a score of 6/10 [17,26,28]. No studies with a high risk of bias were assessed (Figure 2; Table S1).

### 3.4. Results of Synthesis

**Figure 3:**
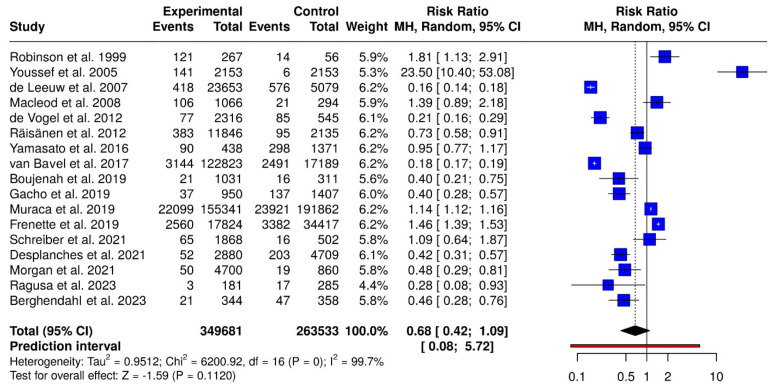
Pooled results of OASIS incidence in nulliparous women who underwent OVD with episiotomy (MLE or ME) versus OVD without episiotomy [8,17,18,19,21,22,23,24,26,27,28,29,30,33,34,36,37].

**Figure 4:**
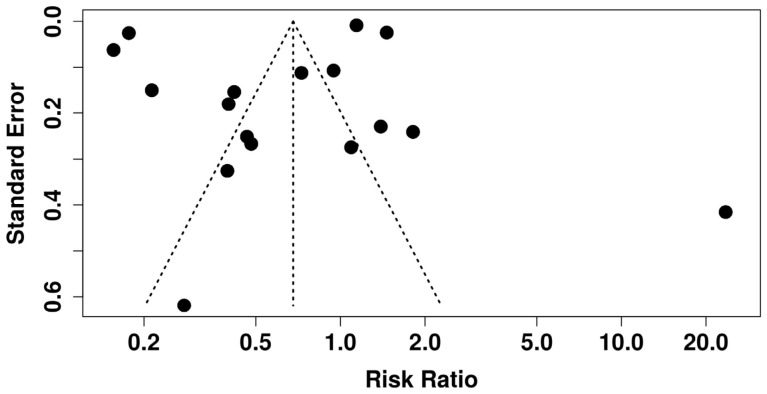
Bias assessment plot of OASIS incidence in nulliparous women who underwent OVD with episiotomy (MLE or ME) versus OVD without episiotomy.

We analyzed a total of 17 studies, involving 349,681 nulliparous women who underwent OVD (VD and FD) with episiotomy (MLE and ME), and 263,533 women in the control cohort who underwent OVD (VD and FD) without episiotomy. Considering the incidence of OASIS, based on the random effects model meta-analysis of RR, there was no statistically significant difference between the two cohorts, with a pooled RR of 0.68 (95% CI of 0.42–1.09) (Figure 3). A significant heterogeneity was detected; the I^2^ value indicated that 99.7% of the variability among studies arose from heterogeneity rather than random chance. The funnel plot (Figure 4) did not indicate a potential publication bias. Egger’s test did not support the presence of funnel plot asymmetry (*p*: 0.293).

**Figure 5:**
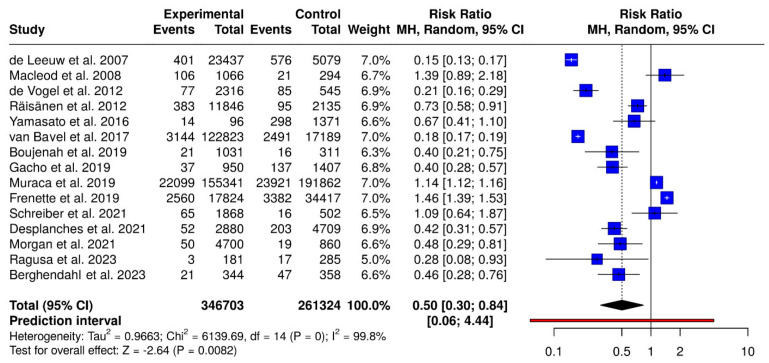
Pooled results of OASIS incidence in nulliparous women who underwent OVD with MLE versus OVD without episiotomy [18,19,21,22,23,24,26,27,28,29,30,33,34,36,37].

**Figure 6:**
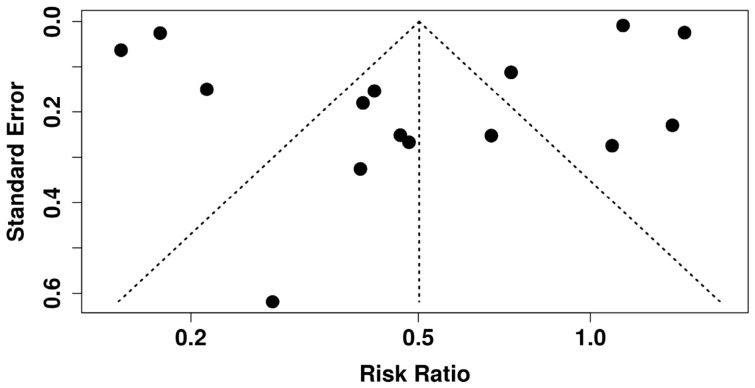
Bias assessment plot of OASIS incidence in nulliparous women who underwent OVD with MLE versus OVD without episiotomy.

We delved deeper into the issue of heterogeneity by focusing the analysis on just 15 studies that analyzed the role of MLE during OVD with a total of 346,703 nulliparous women in the experimental cohort with MLE and 261,324 women in the control cohort without episiotomy. Based on the random effects meta-analysis, there was a statistically significant difference with a lower rate of OASIS in favor of MLE: the pooled RR was 0.5 with a 95% CI of 0.30–0.84 (Figure 5). A significant heterogeneity was still detected (I^2^ = 99.8%). The funnel plot did not indicate a potential publication bias (Figure 6). Egger’s test did not indicate funnel plot asymmetry (*p* = 0.22).

Conversely, a further subgroup analysis, which analyzed the role of ME during OVD compared to a control group without episiotomy, did not show a statistical difference between the two cohorts in terms of OASIS with a pooled RR of 2.21 (95% CI of 0.74–6.59) (Figure S1—Supplementary Material).

**Figure 7:**
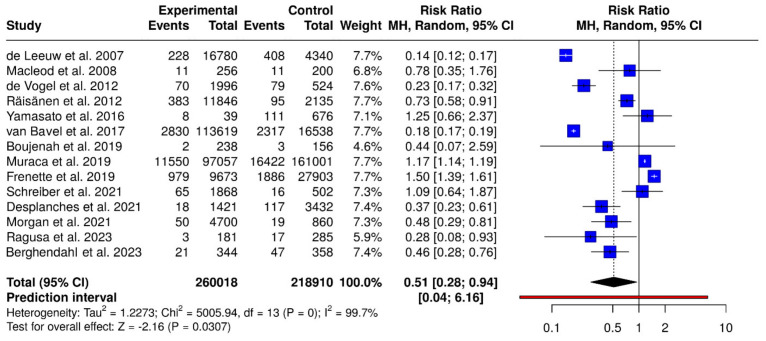
Pooled results of OASIS incidence in nulliparous women who underwent VD with MLE versus VD without episiotomy [18,19,21,22,23,24,27,28,29,30,33,34,36,37].

Furthermore, a subgroup analysis was conducted encompassing 14 studies that focused on the role of MLE during VD. This analysis included 260,018 nulliparous women in the experimental cohort with MLE and 218,910 women in the control cohort without episiotomy. Based on the random effects meta-analysis, there was a statistically significant difference between the two cohorts, with a lower rate of OASIS in favor of MLE: the pooled RR was 0.51 with a 95% CI of 0.28–0.94. A significant heterogeneity was still detected (I^2^ = 99.7%) (Figure 7).

**Figure 8:**
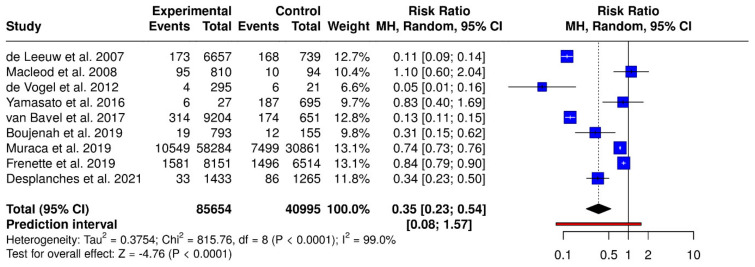
Pooled results of OASIS incidence in nulliparous women who underwent FD with MLE versus FD without episiotomy [18,19,22,23,24,27,28,33,37].

Another subgroup analysis was conducted on 9 studies that considered the role of MLE during FD. A total of 85,654 nulliparous women in the experimental cohort with MLE and 40,995 women in the control cohort without episiotomy were analyzed. Based on the random effects meta-analysis, there was a statistically significant difference between the two cohorts in terms of OASIS, lower in MLE group: the pooled RR was 0.35 with a 95% CI of 0.23–0.54. A significant heterogeneity was still detected (I^2^ = 99.0%) (Figure 8).

Considering data from only three studies, which included multiparous women who underwent MLE during OVD (VD and FD), we did not find a significant difference in terms of the OASIS rate compared to women who did not have an episiotomy. The pooled RR was 0.74 with a 95% CI of 0.17–3.25. The I2 value indicates that 95.7% of the variability among studies arose from heterogeneity rather than random chance (Figure S2—Supplementary Material).

## 4. Discussion

Based on our current knowledge, this study is the first systematic review and meta-analysis in the existing literature to investigate the role of episiotomy (ME and MLE) in more than 2 million women who underwent OVD. The results of our study indicated that the use of MLE in nulliparous women experiencing OVD, encompassing both VD and FD, is associated with a reduced risk of OASIS.

The role of episiotomy during unassisted vaginal delivery has been the subject of extensive debate in recent decades. Nevertheless, the impact of this practice on OVD remains to be elucidated. OVD has been identified as a significant risk factor for OASIS, noted in both VD and FD, with the latter being associated with an even higher risk [39]. A recent trend, supported by data from national population databases, has been observed in several countries showing a decline in the rate of episiotomies during OVD and an increase in the incidence of OASIS cases [40,41,42,43]. Notably, the 2018 WHO recommendations on intrapartum care did not provide specific guidance on the use of episiotomy during OVD. The International Federation of Gynecology and Obstetrics (FIGO) endorse a strictly restrictive global policy, maintaining episiotomies reserved solely for immediate medical indications, such as an unyielding perineum causing failure to progress or acute fetal distress. The American College of Obstetricians and Gynecologists (ACOG) keep a conservative position, recommending against routine episiotomy for all OVDs. However, if episiotomy is necessary, MLE is preferable to ME to avoid rectal extension. The Royal College of Obstetricians and Gynecologists (RCOG) explicitly notes that the protective data supporting a selective MLE against OASIS are significantly stronger for nulliparous women and during forceps extractions. Our results bridge the gap between these varying guidelines by isolating exactly which patient cohort benefits from which intervention. The lack of any significant reduction in OASIS rates among multiparous women in our analysis strongly reinforces the highly restrictive mandates of the WHO and FIGO for this population.

Two other systematic reviews and meta-analyses have previously investigated the morbidity associated with episiotomy in OVD [44,45]. Lund et al. investigated whether the use of MLE or lateral episiotomy influenced the risk of OASIS exclusively in VD among primiparous women, demonstrating a 50% reduction in the risk of OASIS (OR 0.53, 95% CI 0.37–0.77) [44]. The authors of the other study [45] also limited their focus to the role of episiotomy during VD. They found that MLE and ME in parous women may increase the rate of OASIS at VD, whereas lateral episiotomy in nulliparous women could be associated with a decreased risk of OASIS. Nonetheless, two of the more recent large-scale cohort studies were not included in this review [46,47]. Indeed, the above-mentioned reviews and meta-analyses encompassed a time period between 1994 and 2010, during which studies were conducted. Additionally, the methodological approach used in these studies may warrant scrutiny. A third meta-analysis [47] of over 700,000 women evaluated the role of MLE and lateral episiotomy (LE) during operative delivery in reducing the rate of OASIS demonstrating a 40% reduction in the odds of OASIS with MLE/LE use.

The most significant strength of our study is the exhaustive analysis of the available literature up to 2025, conducted through a rigorous methodological process and clearly defined inclusion and exclusion criteria. Furthermore, the methodological quality of the original studies was objectively assessed using the Newcastle–Ottawa Scale. In contrast to previous reviews and meta-analyses, our study considered both ME and MLE across all types of instrumental deliveries (VD and FD). Another major strength is the large cumulative sample size, which enabled multiple subgroup analyses with adequate statistical power. Lastly, the absence of significant publication bias in both the overall and subgroup meta-analyses further reinforces the soundness and reliability of our findings. Despite the considerable heterogeneity among the included studies, the use of MLE in nulliparous women undergoing OVD (VD and/or FD) was associated with a 50% reduction in the risk of OASIS. When stratified by instrument, we further demonstrated that MLE reduces the risk of OASIS by 49% during VD and by 65% during FD in nulliparous women.

Our findings also diverge from those reported by SagiDian et al. [45]. In contrast to their conclusions, there was not an increased risk of OASIS noticed as associated with MLE in multiparous women undergoing OVD, although this result is based on a limited number of studies (*n* = 3). While interpreting this evidence with caution, it nonetheless suggests that MLE does not confer additional harm in this population.

A major source of complexity in interpreting our findings is the substantial heterogeneity across the included studies. Although we have examined this in detail through various subgroup analyses, the heterogeneity remains extremely high in most of the pooled analyses. This likely reflects the multifactorial nature of episiotomy practice, encompassing variations in indications, techniques, and clinical contexts, which inevitably limits the robustness of the conclusions. Furthermore, most of the included studies are retrospective observational cohort studies; therefore, it is not possible to exclude the presence of residual confounding, despite the statistical adjustments applied in the analyses.

In fact, study populations differed considerably with respect to parity, with some cohorts restricted to nulliparous women and others including mixed or predominantly multiparous populations. As parity is one of the strongest predictors of OASIS, differences in its distribution most likely contributed to the observed between-study variation in effect estimates. The subgroup analysis in multiparous women is based on few studies and participants; therefore, conclusions about the absence of benefit in this population should be interpreted with caution.

Furthermore, definitions and diagnostic approaches for OASIS varied across study periods and settings. Earlier studies often relied solely on clinical diagnosis without standardized classification systems, whereas more recent studies have benefited from greater awareness, structured classification, and the use of endoanal ultrasound. Underdiagnosis in older studies and improved detection in contemporary cohorts may therefore partly explain the observed temporal heterogeneity.

Obstetric practice has also evolved substantially over time. The widespread adoption of restrictive episiotomy policies has changed in the preferred mode of OVD (particularly the increased use of vacuum extraction relative to forceps), and improvements in perineal assessment techniques may all have influenced both the baseline risk of OASIS and the relative effect of operative delivery and episiotomy on perineal outcomes.

Another important limitation of the included observational studies is the potential for confounding by indication, which warrants careful consideration when interpreting the pooled estimates. Episiotomy is frequently performed in clinical situations already considered to carry an increased risk of severe perineal trauma, such as fetal distress, shoulder dystocia, a rigid perineum, suspected fetal macrosomia, or anticipated difficult operative vaginal delivery. Under these circumstances, the decision to perform an episiotomy is not random but reflects the clinician’s assessment of imminent obstetric risk. Regardless of the procedure itself, women who undergo episiotomy may differ systematically from those who do not. This may create bias whereby episiotomy appears to be associated with higher rates of OASIS simply because it is more often performed in complex or high-risk births. Although several studies attempted to adjust for known confounders, residual confounding is likely to persist and ought to be taken into account when considering cause-and-effect relationship.

Other factors may contribute to the heterogeneity observed across the included studies: substantial variability in methodological design, differences in maternal characteristics such as age and BMI, variation in episiotomy rates, and differences in the angle at which MLE was performed. Leading obstetric and gynecologic societies recommend performing MLE at a 60° angle from the midline at the time of crowning, in order to achieve a post-delivery angle between 40° and 60° [9]. However, accurately identifying the precise moment of crowning when episiotomy is typically performed is challenging in clinical practice and is frequently underestimated. This technical limitation likely contributes to variability in the actual post-delivery angle and, consequently, to the heterogeneity in reported outcomes.

One of the main limitations of this systematic review and meta-analysis is the limited availability of evidence from RCTs. Among the included studies, only one randomized controlled trial was identified: the Swedish EVA trial by Berghendahl et al. [36], which demonstrated that lateral episiotomy significantly reduced the risk of OASIS during vacuum-assisted delivery. These findings are consistent with those of a previous emulated target trial based on Swedish registry data, which also reported a protective effect of lateral episiotomy in vacuum-extraction deliveries [48]. However, both studies were conducted within the Swedish obstetric setting, characterized by relatively high baseline rates of OASIS, restrictive but protocolized use of lateral episiotomy, and intensive postpartum perineal assessment. Therefore, the generalizability of these findings to other healthcare systems and obstetric practices may be limited. Furthermore, even in randomized or quasi-experimental settings, episiotomy tends to be used more frequently in technically difficult OVDs, and the clinical context in which the intervention is applied may continue to influence outcomes. Hence, some degree of residual confounding and context-specific risk cannot be entirely excluded.

As stated by Sultan et al. [9], “episiotomy is not a treatment for OASIS but rather a risk-modifying factor.” In this context, the optimal approach may be to meticulously conduct designed observational studies capable of comprehensively assessing the impact of specific risk factors. Moreover, recruiting women to undergo episiotomy during OVD is challenging and not always ethically acceptable, further limiting the feasibility of RCTs in this field.

A further important limitation of our review is the inability to assess differences in postpartum pain, urinary incontinence, and fecal incontinence due to the insufficient and highly inconsistent reporting of these outcomes across the included studies. This substantial gap in the available evidence highlights a critical need for future research to adopt standardized and patient-centered outcome measures, without which meaningful comparisons and robust conclusions remain unattainable.

## 5. Conclusions

Our systematic review and meta-analysis highlights a critical and nuanced distinction in the utility of episiotomy during OVD, challenging the widespread trend away from its routine use in instrumental births. While pooled data for all episiotomy techniques, ME and MLE, demonstrated no overall difference in OASIS rates among nulliparous women, a distinct protective effect emerged when MLE was analysed separately. Specifically, the use of MLE during both vacuum and forceps deliveries in nulliparous women was associated with a statistically significant reduction in severe perineal trauma. Notably, this protective effect was not observed in multiparous women, for whom OASIS rates remained unaffected by episiotomy status.

These findings prompt a re-evaluation of current episiotomy practices in the context of instrumental deliveries. Although episiotomy constitutes an intentional perineal injury, this acute intervention must be weighed against the potentially profound and long-term consequences of OASIS, including anal incontinence, chronic pain, and sexual dysfunction. In cases where instrumental delivery increases the risk of severe perineal trauma in a previously intact perineum, the selective use of MLE may offer a meaningful harm-reduction strategy, with benefits that outweigh the morbidity associated with the incision itself.

At the same time, clinical translation of these findings requires caution. The predominance of retrospective data introduces a risk of confounding by indication, and the substantial heterogeneity across included studies limits the ability to draw definitive causal conclusions. Thus, while our analysis provides compelling evidence supporting a protective role for MLE in nulliparous women undergoing OVD, it reinforces the importance of individualized clinical decision-making rather than uniform application of episiotomy.

To address remaining uncertainties, optimize patient outcomes, and better understand the sources of heterogeneity, large-scale prospective studies are needed. Such investigations would allow for more robust control of confounding variables and help clarify the specific clinical contexts in which MLE confers the greatest benefit.

## Figures and Tables

**Table 1:** Characteristics of the studies and basic characteristics of the patients included in the systematic review and meta-analysis.

Authors	Year	Sample Size	Study Design	Mono/ Multi Center	Parity	BMI kg/m^2^	Objective’s Study	Outcome Definition
Ecker J.L., et al. [16]	1997	2041	Retrospective	Mono	NU MU	NS	To examine the association between maternal vaginal and perineal morbidity and epi performed at OVD	The rate of vaginal and 3rd/4th-degree lacerations
Robinson J.N. et al. [17]	1999	1942	Retrospective	Mono	NU	NS	To determine whether choice of obstetric instrument at OVD is associated with any differences in the rate of significant perineal trauma and whether this rate is modified by the use of epi	The occurrence of significant perineal trauma, compared with spontaneous vaginal deliveries during the same period.
Youssef R. et al. [18]	2005	2153	Retrospective	Mono	NU: 53.4% (1150) MU: 46.6% (1003)	NS	To investigate the maternal and neonatal morbidity related to use of epi for VD and FD	The rate of 3rd/4th-degree and shoulder dystocia
de Leeuw J.W. et al. [19]	2007	28,732	Retrospective	Multi	VD: NU: 81.2% (17,263) MU: 18.8% (3991)	NS	To determine the risk factors for OASIS during OVD	Individual obstetric factors, e.g., no epi, mediolateral and midline epi, etc.
					FD: NU: 85.7% (6408) MU: 14.3% (1070) *p* < 0.001			
Macleod M. et al. [33]	2008	1360	Prospective	Multi	NU	>30 17.3%	To evaluate the maternal and neonatal morbidity of OVD in relation to the use of epi	The primary outcome was anal sphincter tearing (3rd/4th degree). Secondary outcomes included postpartum haemorrhage, neonatal trauma and pelvic floor symptoms up until 10 days postpartum
Murphy D.J. et al. [35]	2008	317	Pilot RCT	Multi	NU	>30: 13.8% (44/200)	To compare the maternal and neonatal outcomes of OVD in relation to the use of epi	The primary outcome was anal sphincter tearing (3rd/4th degree). Secondary outcomes included postpartum haemorrhage, neonatal trauma and pelvic floor symptoms up until 10 days postpartum
Revicky V. et al. [20]	2010	10,314	Retrospective	Multi	NU: 46% (4741) MU: 54% (5573)	NS	To analyse the significance of risk factors and the role of epi in preventing OASIS at vaginal delivery	First a univariate analysis was done to identify factors that had a significant association with OASIS. This was followed by an analysis of two multivariate logistic regression models
de Vogel J. et al. [21]	2012	2861	Retrospective	Multi	NU: with epi 87.6% (2026) no epi 73.5% (399) *p* < 0.001	NS	To evaluate the frequency of OASIS in women undergoing OVD and to assess whether a mediolateral epi is protective for developing OASIS in these deliveries	The primary outcome was rate of OASIS
					MU: with epi 12.4% (288) no epi 26.5.5% (144) *p* < 0.001			
Räisänen S. et al. [22]	2012	16,802	Retrospective	Multi	NU: 77.3% (12,985) MU: 22.7% (2821)	NU: with OASIS 23.4 ± 4.1 no OASIS 23.6 ± 4.4; *p* = 0.38	To identify and quantify the risks of OASIS separately in NU, including women admitted for a first vaginal delivery after a previous caesarean section for their first birth, and MU delivered by vacuum extraction in Finland where the type of epi is exclusively lateral	The primary outcome was the rate of OASIS
						MU: with OASIS 25.0 ± 4.8 no OASIS 24.6 ± 4.7 *p* = 0.57		
Yamasato K et al. [23]	2016	22,800	Retrospective	Mono	NU: with epi 48.3% (733) no epi 29.2% (6135) *p* < 0.001	with epi 29.26 ± 5.41 no epi 30.83 ± 6.26 *p* = 0.0001	To examine maternal and neonatal injuries with restricted epi use	The primary outcome was maternal injury (3rd/4th-degree lacerations) with neonatal injury as a secondary outcome
van Bavel J. et al. [24]	2017	170,969	Retrospective	Multi	NU: with epi 85% (122,823) no epi 65% (17,189) *p* < 0.001	NS	To assess whether MLE is associated with a reduction in the rate of OASIS during OVD	The primary outcome was OASIS in women with or without MLE in both types of OVD
					MU: with epi 15% (21,716) no epi 35% (9241) *p* < 0.001			
Boujenah J. et al. [8]	2018	1342	Retrospective	Mono	NU	with epi 24 ± 4.6 no epi 24 ± 5.2	To compare the OASIS rate during OVD according to epi practice in a current French modern obstetrical cohort of NU women	The primary outcome measure was 3rd/4th-degree tears
Gonzalez-Díaz E. et al. [25]	2018	938	Retrospective	Mono	NU MU	NS	To analyse the effect of MLE and perineum characteristics on the occurrence of OASIS in OVD	The primary outcome was the rate of OASIS
Gacho B. et al. [26]	2019	2357	Retrospective	Mono	NU: 79.8% (1870) MU: 21.2% (472)	23 ± 4.6	To investigate whether the implementation of a restrictive episiotomy policy in operative deliveries changes the incidence of OASIS	The primary outcome was the evolution of the OASI and MLE rates. The secondary outcome was the occurrence of OASI during OVD with or without MLE
Muraca G.M. et al. [27]	2019	1,980,273	Retrospective	Multi	NU: 44.4% (877,676) MU: 55.5(1,099,597)	NS	To quantify the association between epi and OASIS	The primary outcome was the rate of OASIS
Frenette P. et al. [28]	2019	52,241	Retrospective	Multi	NU MU	NS	To describe associations between epi at the time of FD or VD and OASIS	The primary outcome was the diagnosis of a 3rd/4th-degree perineal laceration as recorded in the delivery record
Schreiber H. et al. [29]	2020	2370	Retrospective	Mono	NU	with epi 23.0 ± 4.5 no epi 23.8 ± 4.6 *p* = 0.164	To evaluate whether epi during VD leads to fewer 3rd/4th-degree tears	The primary outcome was the rate of 3rd/4th-degree perineal tear. Secondary outcomes were other maternal complications, and low neonatal cord pH and Apgar scores
Desplanches T. et al. [37]	2022	7589	Retrospective	Multi	NU	with epi <18.5 7.4% (183) 18.5–24.9 65% (1597) 25–29.9 18.7% (456) ≥30 8.5% (207)	To investigate the associations between MLE and both OASIs and neonatal outcomes, using propensity scores	The primary outcome was the rate of risk of OASIS and neonatal outcome
						no epi <18.5 7.2% (314) 18.5–24.9 64% (2774) 25–29.9 18.7% (812) ≥30 10.3% (448)		
						*p* = 0.01		
Morgan R. et al. [30]	2022	39,487	Retrospective	Mono	NU: 37.4% (14,763) MU: 62.6% (24,724)	<18.5 9.1% (3589) ≥18.5<25 54% (21,197) ≥25 <30 19.8% (7830) ≥30 <35 7.1% (2785) ≥35 <40 2.1% (814) ≥40 0.6% (253)	To evaluate the association between epi and OASIS rates according to the classification’s subgroups	The primary outcome was the overall epi rate in the institution and its trend over time as well as in each subgroup of obstetrical population classification. Secondary outcome was the rate of 3rd/4th degree, and its association with epi practice
Perrin A. et al. [31]	2022	12,346	Retrospective	Mono	NU	with epi 22.18 no epi 22.42	To assess the association between episiotomy and OASIS in nulliparous women at term according to the use of an instrument for delivery with control confounding by indication	The primary outcome was the occurrence of OASIS
						*p* < 0.002		
Evangelopoulos N. et al. [32]	2023	219	Prospective	Multi	NU	23.7 epi angle <45° 23.68 epi angle >45° 23.71 *p* = 0.96	To assess if an epi suture angle >45° from the median line would be associated with a lower risk for OASIs at the time of OVD	The primary objective was the rate of OASIS. Secondary objective, was to explore if there was a possible independent association between epi length and OASIs
Ragusa A. et al. [34]	2023	498	Prospective	Multi	NU: with epi 165 (91.2) no epi 217 (76.1)	with epi <18.5 2.2% (4) <25.0 31.5% (57) <30.0 40.9% (74) ≥30 25.4% (46)	To evaluate the role of epi during OVD with the VD and its correlation with OASIS	The primary outcome was the occurrence of OASIS in VD
					MU: with epi 16 (8.8) no epi 68 (23.9)	no epi <18.5 3.2% (9) <25.0 33% (94) <30.0 40% (114) ≥30 68 23.8% (68)		
					*p* ≤ 0.001.			
Berghendahl S. et al. [36]	2023	717	RCT	Multi	NU	with epi 23.7 (17.7–50.1)	To assess the effect of lateral epi, compared with no epi, on OASIS in NU women requiring vacuum extraction	The primary outcome of the epi in VD trial was OASIS, clinically diagnosed by combined visual inspection and digital rectal and vaginal examination
						no epi 23.7 (16.6–44.5)		

Epi: episiotomy; MU: multiparous; NS: not specified; NU: nulliparous.

**Table 2:** Outcome measures of the studies included in the systematic review and meta-analysis.

Authors	Type of Delivery	Type of Episiotomy	Rate of Episiotomy	Rate of OASIS	Results
Ecker J.L., et al. [16]	VD: 976 (47.8%) FD: 1065 (52.2%)	ME: 97% MLE: 3%	NU: 38.7% MU: 20%	*3rd-degree* NU: 29.7%; MU: 13.3% VD: 22.9%; FD: 29.7%	The epi rate was directly correlated with the rate of 4th-degree laceration (R^2^ = 0.46, *p* = 0.02) but was not significantly correlated with the rate of 3rd-degree lacerations (R^2^ = 0.02, *p* = 0.66) and was inversely correlated with the rate of vaginal laceration (R^2^ = 0.86, *p* = 0.0001)
				*4th-degree* NU: 5.8%; MU: 3.3% VD: 5.5%; FD: 5.3%	
Robinson J.N., et al. [17]	ND: 1619 (83.4%) VD: 161 (8.3%) FD: 162 (8.3%)	ME: 97% MLE: 3%	Overall 47.6% (924/1942)	*Overall delivery* with epi: 23.8% (220/924) no epi: 5.5% (56/1018)	There was a higher rate of 3rd- or 4th-degree laceration when an epi was performed at ND (15.1% vs. 4.4%; *p* = 0.001) and at VD (34.9% vs. 9.4%; *p* = 0.005). In contrast, when FD were used, there was no significant difference in the rate of 3rd- or 4th-degree lacerations when epi was performed (55.1% vs. 45.8%; *p* = 0.4) FD with epi (OR, 15.8; 95% CI, 3.4–73.6) and FD with no epi (OR, 11.0; 95% CI, 1.9–62.7) were associated with a >10-fold increase in significant perineal trauma with respect to VD without epi. VD with epi was associated with a 7-fold increase in significant perineal trauma with respect to VD alone (OR, 6.8; 95% CI, 1.4–31.8)
				ND: 8.7% (141/1619): with epi 15.1% (99/657) no epi 4.4% (42/962)	
			ND: 40.6% (657/1619) VD: 80.1% (129/161) FD: 85.2% (138/162)	VD: 29.8% (48/161 with epi 34.9% (45/129) no epi 9.4% (3/32)	
				FD: 53.7% (87/162) with epi 55.1% (76/138) no epi 45.8% (11/24)	
Youssef R., et al. [18]	VD: 619 (29%) FD:1534 (71%).	>ME (but NS)	VD: 71.4% (442/619) FD: 95.8% (1470/1534)	*Overall OVD* with epi: 6.5% (141/2153) no epi: 0.3% (6/2153)	VD was associated with less use of epi compared with FD (OR 0.10, 95% CI 0.07–0.14). Extensive perineal tears were more likely with use of epi (7.5% vs. 2.5%, adj OR 2.92, 95% CI 1.27–6.72) as was neonatal trauma (6.0% vs. 1.7%, adj OR 2.62, 95% CI 1.05–6.54). Use of epi did not reduce the risk of shoulder dystocia (6.9% vs. 4.6%, adj OR 1.43, 95% CI 0.74–2.76)
				VD: 2.1% (13/616) with epi 1.6% (10/616) no epi 0.5% (3/616)	
				FD: 8.9% (134/1505) with epi 8.7% (131/1505) no epi 0.2% (3/1505)	
de Leeuw J.W. et al. [19]	VD: 21,254 (74%) FD: 7478 (26%)	ME: 0.75% MLE: 81.5%	ME: VD: 0.6% (134/28,732) FD: 1.1% (82/28,732)	VD: 3% (646/21,254) With MLE 1.4% (228/16,780) With ME 7.5% (10/134) no epi 1.4% (408/4340)	MLE protected significantly for anal sphincter damage in both VD (OR 0.11, 95% CI 0.09–0.13) and FD (OR 0.08, 95% CI 0.07–0.11)
			MLE: VD: 78.9% (16,780/28,732) FD: 89% (6657/28,732)	FD: 4.7% (348/7478) With MLE 0.1% (173/6657) With ME 8.5% (7/82) no epi 22.7% (168/739)	
Macleod M. et al. [33]	VD: 456 (33.5%) FD: 904 (66.5%)	Theoretically MLE but not able to truly assess the angle of incision	MLE: VD: 56.1% (256/456)	*Overall OVD* with epi 9.9% (106/1066) no epi 7.1% (21/294)	OASIS rates were not statistically different with use of epi compared with no epi (9.9 vs. 7.1%, add OR 1.11,95% CI 0.66–1.87). Epi use was associated with higher rates of postpartum haemorrhage (28.5 vs. 18.4%, add OR 1.72, 95% CI 1.21–2.45), need for moderate or strong analgesia (90.5 vs. 67.6%, add OR 3.70, 95% CI 2.60–5.27), perineal infection (5.1 vs. 1.4%, add OR 4.04, 95% CI 1.44–11.37) and neonatal trauma (38.1 vs. 22.0%, add OR 1.65, 95% CI 1.20–2.27). Use of epi did not reduce the risk of shoulder dystocia (3.5 vs. 1.7%, add OR 1.42, 95% CI 0.53–3.85)
				VD: 33.5% (456/1360) with epi 4.3% (11/256) no epi 5.5% (11/200)	
			FD: 89.6% (810/904)	FD: 66.5% (904/1360) with epi 11.7% (95/810) no epi 10.6% (10/94)	
Murphy D.J. et al. [35]	ND: 32 (10%) VD: 80 (25.3%) RCT(47) FD: 205 (64.7%) RCT(128)	NS	Women randomised to restrictive use of epi VD: 17%(4/23) FD: 64% (43/67)	RCT: *Overall OVD* routine 8.1% (8/175) vs. restrictive 10.9% (11/175)	Routine use of epi was not associated with a statistically significant difference in OASIS, although the incidence was slightly lower than in the restrictive group (8 of 99 [8.1%] versus 11 of 101 [10.9%], OR 0.72, 95% CI 0.28–1.87)
				VD: 26.8% (47/175) routine 4.2% (2/47) vs. restrective 0% (0/47)	Fewer women randomised to restrictive use of epi with VD received an epi (4/23, 17%) compared with FD where more than half of those randomised to restrictive use received an epi (43/67 64%)
				FD: 73.2% (128/175) routine 9.8% (6/128) vs. restrective 16.4% (11/128)	
Revicky V. et al. [20]	ND: 8472 (82.1%) VD: 1112 (10.8%) FD: 730 (7.1%)	MLE	16.2%(1666/10,314)	*Overall OVD* 3.2% (332/10,314)	The frequency of OASIS was 3.2%. There were statistically significant associations between an increased incidence of OASIS and parity, birth weight, method of delivery and shoulder dystocia. Women giving birth without a MLE were 1.4 times more likely to experience OASIS (95% CI 1.021–1.983)
				ND: 2.3% (194/8472) VD: 6% (66/1112) FD: 9.9% (72/730)	
Joey de Vogel, M.D. et al. [21]	VD: 2520 (88%) FD: 316 (11%) VD + FD: 25 (1%)	MLE	80.9% (2316/2861)	*Overall OVD* with epi 3.5% (77/2316) vs. no epi 15.6% (85/545)	The frequency of OASIS was 5.7%. Women with a MLE were at significantly lower risk for OASIS compared with the women without a MLE in case of an OVD (ad-OR, 0.17; 95% CI, 0.12– 0.24)
			VD: 86.2% (1996/2316) FD: 12.8% (295/2316)	VD: 2520 with epi 3.5% (70/1996) no epi 15.1% (79/524)	
				FD: 316 with epi 1.4% (4/295) no epi 20.6% (6/21)	
Räisänen S. et al. [22]	VD	Lateral	78.5% (12,378/15,806)	Overall OVD with epi 3.2% (412/13,178) no epi 3% (107/3583)	Lateral epi was associated with 46% decreased incidence of OASIS (add OR 0.54, 95% CI 0.42–0.70) in nulliparae delivered by VD. There was no statistically significant association for multiparous women
			NU: 84.9% (11,031/12,985) 1st delivery after a CS for the 1st birth 82.6% (823/996)	NU: 13,981 with epi 3.2% (383/11,846) no epi 4.4% (95/2135) *p* = 0.01	
			MU: 47.9% (1331/2780)	MU: 2780 with epi 2.2% (29/1332) no epi 0.8% (12/1448) *p* = 0.002	
Yamasato K. et al. [23]	ND: 21,021 (92.2%) VD: 913 (4%) FD: 866 (3.8%)	ME: 87.8%	Overall 6.7% (1529/22,800)	Overall OVD with MLE 14.5% (14/96) with ME 22.2% (76/342) No epi 21.7% (298/1371)	Epi continues to be associated with increased third- and 3rd/4th degree lacerations with restricted use, particularly in spontaneous vaginal deliveries
		MLE: 12.8%	VD: 26.0% (237/913) FD: 19.7% (171/866)	VD: with MLE 20.5% (8/39) with ME 21.2% (42/198) No epi 16.4% (111/676)	
				FD: with MLE 23.1% (6/27) with ME 23.4% (34/144) No epi 26.9% (187/695)	
van Bavel J. et al. [24]	VD + FD: 170,969	MLE: 84.5% (144,539)	VD: NU: 87.3% (113,619/130,157) MU: 69.4% (20,246/29,183)	VD: NU: with epi 2.5% (2830/113,619) no epi 14% (2317/16,538)	The use of a MLE during both vacuum delivery and forceps delivery is associated with a fivefold to tenfold reduction in the rate of OASIS in primiparous and multiparous women
		No epi 26,430	FD: NU: 93.4% (9204/9855) MU: 8.3% (1470/1774)	MU: with epi 2.1% (416/20,246) no epi 7.5% (667/8937)	
				FD: NU: 9855 with epi 3.4% (314/9204) no epi 26.7% (174/651)	
				MU: with epi 2.6% (38/1470) no epi 14.2% (43/304)	
Boujenah J. et al. [8]	VD: 394 (29.3%) FD: 948 (70.7%)	MLE	VD: 60.4% (238/394) FD: 83.6% (793/948)	*Overall OVD* with epi 2% (21/1031) no epi 5.1% (16/311) *p* value < 0.01	Episiotomy is a modifiable risk factor which can contribute to reduce the risk of OASIS in nulliparous women with operative vaginal delivery
				VD: with epi 0.8% (2/238) no epi 2.1% (3/156) NSS	
				FD: with epi 2.4% (19/793) no epi 7.7% (12/155) *p* value < 0.05	
Gonzalez-Díaz E. et al. [25]	VD: 13.8% (129) FD: 44.1% (413)	MLE	Overall OVD 97%	*Overall OVD* 15.7% (150/958)	The main risk factors of OASIs in OVD are nulliparity, persistent occipitoposterior position, birthweight >3500 g and a distance of perineal body ≤30 mm. We also find two protective factors which can be modified by the obstetrician at the time of performing the epi, such as an angle of epi >30 and a distance epi-fourchette >5 mm. Women in the OASIS group were more likely to be NU (95.6% vs. 89.1%, *p* = 0.023)
				VD: 33.5% FD: 51.5%	
Gacho B. et al. [26]	VD: 35.9% (847) FD: 57.3 (1350) Spatula: 6.8% (160)	MLE	Overall OVD 40.3% (950/2357)	*Overall OVD* with epi 3.9% (37/950) no epi 9.7% (137/1407) *p* value < 0.01	OVD with MLE was associated with a three times lower OASIS occurrence than that without MLE (add OR = 0.29, 95% CI [0.20–0.43])
				VD: 847 with epi 1.2% no epi 5.7% OR = 0.19 [0.02–0.74]	
				FD: 1350 with epi 4.1% no epi 13.6% OR = 0.28 [0.17–0.43]	
Muraca G.M. et al. [27]	ND: 2,205,056 VD: 258,058 FD: 89,145	MLE	VD: 37.6% (97,057/258,058)	VD: with epi 11.9% (11,550/97,057) no epi 10.2% (16,422/161,001)	Epi was associated with lower rates of OASIS among FD in nulliparous women (add RR 0.63, 95% CI 0.61–0.66), and women with vaginal birth after cesarean (add RR 0.71,95% CI 0.60–0.85), but not among parous women without a previous cesarean (add RR 1.16, 95% CI 1.00–1.34). Epi was more common than among FD (55.6%) than VD (25.7%)
			FD: 65.4% (58,284/89,145)	FD: with epi 18.1% (10,549/58,284) no epi 24.3% (7499/30,861)	
Frenette P. et al. [28]	VD: 37,576 (71.9%) FD: 14,665 (28.1%)	ME MLE	VD: 25.7% (9673/37,576)	*Overall OVD* with epi 14.4% (2560/17,824) no epi 9.8% (3382/34,417) *p* value < 0.001	Epi was associated with increased odds of severe perineal lacerations for VD among women with (OR 2.48; 95% CI 1.96–3.13) and without (OR 1.12; 95% CI 1.02–1.22) a prior vaginal delivery. Among FD, epi was associated with increased odds of OASIS for those with a previous vaginal delivery (OR 1.52; 95% CI 1.12–2.06), but it was protective for women with no previous vaginal delivery (OR 0.73; 95% CI 0.67–0.79). Midline compared with mediolateral epis increased the odds of OASIS in FD (OR 2.73; 95% CI 2.37–3.13) and VD (OR 1.94; 95% CI 1.65–2.28)
			FD: 55.6% (8151/14,665)	VD: with epi 10.1% (979/9673) no epi 6.8% (1886/27,903) *p* value < 0.001	
				FD: with epi 19.4% (1581/8151) no epi 23% (1496/6514) *p* ≤ 0.001	
Schreiber H. et al. [29]	VD	MLE	VD: 79% (1868/2370)	VD with epi 2.74% (65/1868) no epi 3.2% (16/502) *p* = 0.58	Using selective epi for patients delivering vaginally with the assistance of soft cap vacuum does not increase 3th/4th-degree perineal tears
Desplanches T. et al. [37]	VD: 4853 (64.3) FD: 2698 (35.7)	MLE	VD: 49.8% (1421/2880)	*Overall OVD* with epi 1.8% (52/2880) no epi 4.3% (203/4709)	Epi was associated with a lower rate of OASIS in forceps/spatula delivery (2.3 vs. 6.8%, RR 0.38, 95% CI 0.28–0.52) and in VD (1.3 vs. 3.4%, RR 0.27, 95% CI 0.20–0.38) as compared with no epi
			FD: 50.2% (1433/2880)	VD: with epi 1.3% (18/1421) no epi 3.4% (117/3432)	
				FD: with epi 2.3% (33/1433) no epi 6.8% (86/1265)	
Morgan R. et al. [30]	VD: 1780	MLE	NS	VD: NU with epi 1.1% (50/4700) no epi 2.2% (19/860) *p* = 0.05	The analysis in each subgroup showed that the association between OASIS and episiotomy was significant only in Group 2 (Nulliparous women with a single cephalic pregnancy at ≥37 weeks of pregnancy, instrumental delivery; 1.24%) with a decrease in OASIS rate if using episiotomy (OR 0.5; 95% CI 0.3–0.8)
				MU with epi 0.7% (12/1626) no epi 1.2% (11/920) *p* = 0.241	
Perrin A. et al. [31]	OVD: 5560 VD: 560 FD: 4954	MLE	60.2% (4700)	*Overall OVD* with epi 1.1% (50/4700) no epi 2.2% (19/860) *p* = 0.005	After stratification on use of instrument, an association between epi and OASIS was shown in the case of instrumental delivery (OR 0.46, 95% CI 0.26–0.80) but not if the delivery was spontaneous (OR 0.76, 95% CI 0.29–1.98). The result was similar after matching on propensity score (in the case of OVD: OR 0.20, 95% CI 0.10–0.75)
Evangelopoulos N. et al. [32]	VD: 107 FD: 110	MLE (>45° and <45°)	VD: epi > 45° 32 epi < 45° 75	VD: 0.9% (1/107) FD: 3.4% (4/110) *p* = 0.05	This study did not demonstrate a significant reduction in risk for OASIS at the time of OVD when the epi suture angle was >45° from the median line
			FD: epi > 45° 31 epi < 45° 79		
Ragusa A. et al. [34]	VD: 466	MLE	VD: 61.2% (285/466)	with epi 3/181 (1.8%) no epi 17/285 (6.0%) *p* ≤ 0.001	The use of epi during OVD was associated with much lower OASIS rates in nulliparous women with a VD; OR 0.23 (CI 95% 0.07–0.81) *p* = 0.037 in nulliparous women
Bergehndahl S. et al. [36]	VD: 717	MLE	VD: 47.8% (344/717)	with epi 6% (21/344) no epi 13% (47/358) *p* = 0.002	In the intervention group, 21 (6%) of 344 women sustained OASIS, compared with 47 (13%) of 358 women in the comparison group (*p* = 0.002). The risk difference was −7.0% (96% CI −11.7% to −2.5%). The risk ratio adjusted for site was 0.47 (96% CI 0.23 to 0.97) and unadjusted risk ratio was 0.46 (0.28 to 0.78)

VD: vacuum, delivery; FD: focep delivery; NU: nulliparous; MU: multiparous; NS: not specifie.

## Data Availability

The original contributions presented in this study are included in the article/Supplementary Materials. Further inquiries can be directed to the corresponding author(s).

## Supplementary Materials

The following supporting information can be downloaded at: https://www.mdpi.com/article/10.3390/jcm15134962/s1, Figure S1: Pooled results of OASIS incidence in nulliparous women who underwent OVD with ME versus OVD without episiotomy [17,18,19,23]; Figure S2. Pooled results of OASIS incidence in multiparous women who underwent OVD with MLE versus OVD without episiotomy [22,24,30]; Table S1: Detailed Newcastle-Ottawa Scale of each included cohort study [8,16,17,18,19,20,21,22,23,24,25,26,27,28,29,30,31,32,33,34,35,36,37,38].

## Abbreviations

The following abbreviations are used in this manuscript:

ACOG	American College of Obstetricians and Gynecologists
BMI	Body mass index
CI	Confidence Interval
EPI	Episiotomy
FIGO	International Federation of Gynecology and Obstetrics
FD	Forceps delivery
ME	Median episiotomy
MLE	Medio-lateral episiotomy
MU	Multiparous
NOS	Newcastle–Ottawa Scale
NU	Nulliparous
OVD	Operative vaginal delivery
OASIS	Obstetric anal sphincter injuries
RCOG	Royal College of Obstetricians and Gynaecologists
RCT	Randomized control trial
RoB	Risk of bias
RR	Risk ratio
VD	Vacuum delivery
WHO	World Health Organization

## References

[1] Cunningham F.G., Leveno K., Dashe J., Hoffman B., Spong C., Casey B. (2024). Williams Obstetrics.

[2] Wagner M. (1999). Episiotomy: A form of genital mutilation. Lancet.

[3] WHO Technical Working Group (1997). Care in Normal Birth: A Practical Guide.

[4] Carroli G., Belizán J. (1999). Episiotomy for vaginal birth. Cochrane Database of Systematic Reviews.

[5] Carroli G., Mignini L. (2009). Episiotomy for vaginal birth. Cochrane Database of Systematic Reviews.

[6] World Health Organization (2018). WHO Recommendations: Intrapartum Care for a Positive Childbirth Experience.

[7] Jiang H., Qian X., Carroli G., Garner P. (2017). Selective versus routine use of episiotomy for vaginal birth. Cochrane Database Syst. Rev..

[8] Boujenah J., Tigaizin A., Fermaut M., Murtada R., Benbara A., Benchimol M., Pharisien I., Carbillon L. (2019). Is episiotomy worthwhile to prevent obstetric anal sphincter injury during operative vaginal delivery in nulliparous women?. Eur. J. Obstet. Gynecol. Reprod. Biol..

[9] Sultan A.H., Thakar R., Ismail K.M., Kalis V., Laine K., Räisänen S.H., de Leeuw J.W. (2019). The role of mediolateral episiotomy during operative vaginal delivery. Eur. J. Obstet. Gynecol. Reprod. Biol..

[10] Page M.J., Bossuyt P.M., Boutron I., Hoffmann T.C., Mulrow C.D., Shamseer L., Tetzlaff J.M., Akl E.A., Brennan S.E., Chou R. (2021). The PRISMA 2020 statement: An updated guideline for reporting systematic reviews. BMJ.

[11] DerSimonian R., Laird N. (2015). Meta-analysis in clinical trials revisited. Contemp. Clin. Trials.

[12] Higgins J.P., Thompson S.G. (2002). Quantifying heterogeneity in a meta-analysis. Stat. Med..

[13] Higgins J.P., Thompson S.G., Deeks J.J., Altman D.G. (2003). Measuring inconsistency in meta-analyses. BMJ.

[14] Egger M., Davey Smith G., Schneider M., Minder C. (1997). Bias in meta-analysis detected by a simple graphical test. BMJ.

[15] Fekete J.T., Győrffy B. (2025). MetaAnalysisOnline.com: Web-based tool for the rapid meta-analysis of clinical and epidemiological studies. J. Med. Internet Res..

[16] Ecker J.L., Tan W.M., Bansal R.K., Bishop J.T., Kilpatrick S.J. (1984). Is there a benefit to episiotomy at operative vaginal delivery? Observations over ten years in a stable population. Am. J. Obstet. Gynecol..

[17] Robinson J.N., Norwitz E.R., Cohen A.P., McElrath T.F., Lieberman E.S. (1999). Episiotomy, operative vaginal delivery, and significant perineal trauma in nulliparous women. Am. J. Obstet. Gynecol..

[18] Youssef R., Ramalingam U., Macleod M., Murphy D.J. (2005). Cohort study of maternal and neonatal morbidity in relation to use of episiotomy at instrumental vaginal delivery. BJOG.

[19] De Leeuw J.W., De Wit C., Kuijken J.P., Bruinse H.W. (2008). Mediolateral episiotomy reduces the risk for anal sphincter injury during operative vaginal delivery. BJOG.

[20] Revicky V., Nirmal D., Mukhopadhyay S., Morris E.P., Nieto J.J. (2010). Could a mediolateral episiotomy prevent obstetric anal sphincter injury?. Eur. J. Obstet. Gynecol. Reprod. Biol..

[21] De Vogel J., van der Leeuw-van Beek A., Gietelink D., Vujkovic M., de Leeuw J.W., van Bavel J., Papatsonis D. (2012). The effect of a mediolateral episiotomy during operative vaginal delivery on the risk of developing obstetrical anal sphincter injuries. Am. J. Obstet. Gynecol..

[22] Räisänen S., Vehviläinen-Julkunen K., Cartwright R., Gissler M., Heinonen S. (2012). Vacuum-assisted deliveries and the risk of obstetric anal sphincter injuries: A retrospective register-based study in Finland. BJOG.

[23] Yamasato K., Kimata C., Huegel B., Durbin M., Ashton M., Burlingame J.M. (2016). Restricted episiotomy use and maternal and neonatal injuries: A retrospective cohort study. Arch. Gynecol. Obstet..

[24] Van Bavel J., Hukkelhoven C.W.P.M., De Vries C., Papatsonis D.N., de Vogel J., Roovers J.P.W., Mol B.W., de Leeuw J.W. (2018). The effectiveness of mediolateral episiotomy in preventing obstetric anal sphincter injuries during operative vaginal delivery: A ten-year analysis of a national registry. Int. Urogynecol. J..

[25] Gonzalez-Díaz E., Fernández Fernández C., Gonzalo Orden J.M., Fernández Corona A. (2019). Characteristics of the episiotomy and perineum associated with lower risk of obstetric anal sphincter injury in instrumental deliveries. Eur. J. Obstet. Gynecol. Reprod. Biol..

[26] Gachon B., Fradet Menard C., Pierre F., Fritel X. (2019). Does the implementation of a restrictive episiotomy policy for operative deliveries increase the risk of obstetric anal sphincter injury?. Arch. Gynecol. Obstet..

[27] Muraca G.M., Liu S., Sabr Y., Lisonkova S., Skoll A., Brant R., Cundiff G.W., Stephansson O., Razaz N., Joseph K.S. (2019). Episiotomy use among vaginal deliveries and the association with anal sphincter injury: A population-based retrospective cohort study. Can. Med. Assoc. J..

[28] Frenette P., Crawford S., Schulz J., Ospina M.B. (2019). Impact of episiotomy during operative vaginal delivery on obstetrical anal sphincter injuries. J. Obstet. Gynaecol. Can..

[29] Schreiber H., Mevorach N., Sharon-Weiner M., Farladansky-Gershnabel S., Shechter Maor G., Biron-Shental T. (2021). The role of mediolateral episiotomy during vacuum-assisted vaginal delivery with soft cup devices. Arch. Gynecol. Obstet..

[30] Morgan R., Korb D., Sibony O. (2022). Classification and evaluation of episiotomy practices from 2004 to 2020 and association with OASIS. Int. J. Gynecol. Obstet..

[31] Perrin A., Korb D., Morgan R., Sibony O. (2023). Effectiveness of episiotomy to prevent OASIS in nulliparous women at term. Int. J. Gynecol. Obstet..

[32] Evangelopoulos N., Duraes M., Cayrac M., Galtier F., Fritel X., Gachon B., De Tayrac R. (2024). Episiotomy practice in France and prevention of high-grade perineal tears at the time of operative vaginal delivery: A prospective multicentre ancillary cohort study. Int. Urogynecol. J..

[33] Macleod M., Strachan B., Bahl R., Howarth L., Goyder K., Van de Venne M.A., Murphy D.J. (2008). A prospective cohort study of maternal and neonatal morbidity in relation to use of episiotomy at operative vaginal delivery. BJOG.

[34] Ragusa A., Ficarola F., Svelato A., De Luca C., D’Avino S., Carabaneanu A., Ferrari A., Cundari G.B., Angioli R., Manella P. (2023). Is an episiotomy always necessary during an operative vaginal delivery with vacuum? A longitudinal study. J. Matern. Neonatal Med..

[35] Murphy D.J., Macleod M., Bahl R., Goyder K., Howarth L., Strachan B. (2008). A randomised controlled trial of routine versus restrictive use of episiotomy at operative vaginal delivery: A multicentre pilot study. BJOG.

[36] Berghendahl S., Jonsson M., Hesselman S., Ankarcrona A., Leijonhufvud A., Wihlbäck A., Wallström T., Rydström E., Friberg H., Kopp Kallner H. (2024). Lateral episiotomy or no episiotomy in vacuum-assisted delivery in nulliparous women (EVA): Multicentre, open-label, randomised controlled trial. BMJ.

[37] Desplanches T., Marchand-Martin L., Szczepanski E.D., Ruillier M., Cottenet J., Semama D., Simon E., Quantin C., Sagot P. (2022). Mediolateral episiotomy and risk of obstetric anal sphincter injuries and adverse neonatal outcomes during operative vaginal delivery in nulliparous women: A propensity-score analysis. BMC Pregnancy Childbirth.

[38] Wells G.A., Shea B., O’Connell D., Peterson J., Welch V., Losos M., Tugwell P. The Newcastle-Ottawa Scale (NOS) for Assessing the Quality of Nonrandomised Studies in Meta-analyses.

[39] Halle T.K., Salvesen K.Å., Volløyhaug I. (2016). Obstetric anal sphincter injury and incontinence 15–23 years after vaginal delivery. Acta Obstet. Gynecol. Scand..

[40] Blondel B., Alexander S., Zeitlin J., Euro-Peristat Scientific Committee (2016). Variations in rates of severe perineal tears and episiotomies in 20 European countries: A study based on routine national data in the Euro-Peristat Project. Acta Obstet. Gynecol. Scand..

[41] Lallemant M., Bartolo S., Ghesquiere L., Rubod C., Ruffolo A.F., Kerbage Y., Chazard E., Cosson M. (2024). Midterm complications after primary obstetrical anal sphincter injury repair in France. BMC Pregnancy Childbirth.

[42] Lallemant M., Ruffolo A.F., Kerbage Y., Garadebian C., Ghesquiere L., Rubod C., Cosson M. (2024). Clinical practices in the management and follow-up of obstetric anal sphincter injuries: A comprehensive review. Eur. J. Obstet. Gynecol. Reprod. Biol..

[43] Serati M., Ruffolo A.F., Scancarello C., Braga A., Salvatore S., Ghezzi F. (2023). When does oasis cause de novo pelvic floor dysfunction? role of the surgeon’s skills. Int. Urogynecol. J..

[44] Lund N.S., Persson L.K., Jangö H., Gommesen D., Westergaard H.B. (2016). Episiotomy in vacuum-assisted delivery affects the risk of obstetric anal sphincter injury: A systematic review and meta-analysis. Eur. J. Obstet. Gynecol. Reprod. Biol..

[45] Sagi-Dain L., Sagi S. (2015). Morbidity associated with episiotomy in vacuum delivery: A systematic review and meta-analysis. BJOG.

[46] Räisänen S., Selander T., Cartwright R., Gissler M., Kramer M.R., Laine K., Heinonen S. (2014). The association of episiotomy with obstetric anal sphincter injury—Population based matched cohort study. PLoS ONE.

[47] Okeahialam N.A., Wong K.W., Jha S., Sultan A.H., Thakar R. (2022). Mediolateral/lateral episiotomy with operative vaginal delivery and the risk reduction of obstetric anal sphincter injury (OASI): A systematic review and meta-analysis. Int. Urogynecol. J..

[48] Ankarcrona V., Zhao H., Jacobsson B., Brismar Wendel S. (2021). Obstetric anal sphincter injury after episiotomy in vacuum extraction: An epidemiological study using an emulated randomised trial approach. BJOG Int. J. Obstet. Gynaecol..

