# Supplementary material for: Episiotomy in Operative Vaginal Delivery Reduces the Risk of Obstetric Anal Sphincter Injuries in Nulliparous Women: A Systematic Review and Meta-Analysis
Source: J Clin Med. 2026 Jun 25;15(13):4962. doi: 10.3390/jcm15134962 (PMC13362376; doi:10.3390/jcm15134962)
Supplement: Supplementary file 1 [file jcm-15-04962-s001.zip › Supplementary material.pdf]

**Supplementary material**

**Figure S1.** **Pooled results of OASIS incidence in nulliparous women who underwent OVD with ME versus OVD without episiotomy**

| Study | Experimental Events | Experimental Total | Control Events | Control Total | Weight | Risk Ratio MH, Random, 95% CI |
|---|---|---|---|---|---|---|
| Robinson et al. 1999 | 121 | 267 | 14 | 56 | 25.3% | 1.81 [ 1.13; 2.91] |
| Youssef et al. 2005 | 141 | 2153 | 6 | 2153 | 23.1% | 23.50 [10.40; 53.08] |
| de Leeuw et al. 2007 | 17 | 216 | 576 | 5079 | 25.3% | 0.69 [ 0.44; 1.10] |
| Yamasato et al. 2016 | 76 | 342 | 298 | 1371 | 26.3% | 1.02 [ 0.82; 1.28] |
| **Total (95% CI)** | | **2978** | | **8659** | **100.0%** | **2.21 [ 0.74; 6.59]** |
| **Prediction interval** | | | | | | **[ 0.05; 106.15]** |

Heterogeneity: $Tau^2 = 1.1693$; $Chi^2 = 75.82$, df = 3 ($P < 0.0001$); $I^2 = 96.0\%$
Test for overall effect: Z = 1.42 (P = 0.1542)

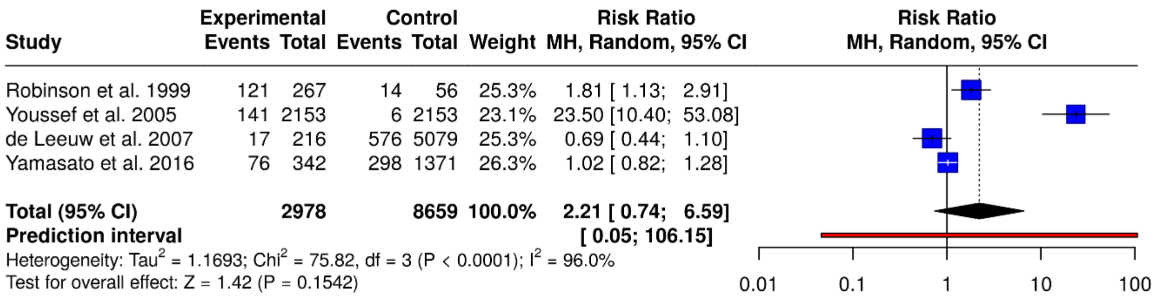


**Figure S2.** **Pooled results of OASIS incidence in multiparous women who underwent OVD with MLE versus OVD without episiotomy**

| Study | Experimental Events | Experimental Total | Control Events | Control Total | Weight | Risk Ratio MH, Random, 95% CI |
|---|---|---|---|---|---|---|
| Räisänen et al. 2012 | 29 | 1332 | 12 | 1448 | 32.9% | 2.63 [1.35; 5.13] |
| van Bavel et al. 2017 | 454 | 21716 | 710 | 9241 | 35.2% | 0.27 [0.24; 0.31] |
| Morgan et al. 2021 | 12 | 1626 | 11 | 920 | 31.9% | 0.62 [0.27; 1.39] |
| **Total (95% CI)** | | **24674** | | **11609** | **100.0%** | **0.74 [0.17; 3.25]** |
| **Prediction interval** | | | | | | **[0.00; 418.37]** |

Heterogeneity: $Tau^2 = 1.6001$; $Chi^2 = 46.39$, df = 2 ($P < 0.0001$); $I^2 = 95.7\%$
Test for overall effect: Z = -0.39 (P = 0.6953)

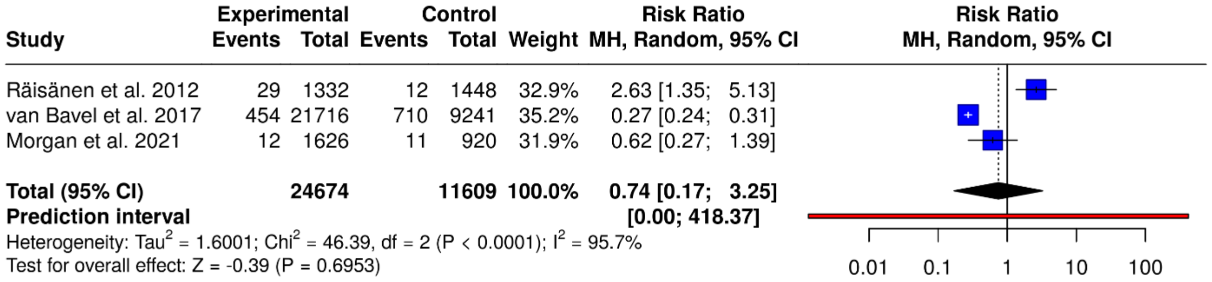

**Table S1. Detailed Newcastle-Ottawa Scale of each included cohort study [48].**

| Study | Selection | | | | Comparability | | Outcome | | | Total quality score |
|---|---|---|---|---|---|---|---|---|---|---|
| | Representativeness of the intervention cohort | Selection of the non-ntervention cohort | Ascertainment of intervention | Demonstration that outcome of interest was not present at start of study | Adjustment for the most important risk factors | Adjustment for other risk factors | Assessment of outcome | Follow-up length | Loss to follow-up rate | |
| Ecker JL et al, 1997 | ★ | 0 | 0 | ★ | ★ | 0 | ★ | 0 | ★ | **5★** |
| Robinson JN et al. 1999 | ★ | ★ | 0 | ★ | ★ | 0 | ★ | 0 | ★ | **6★** |
| Youssef R et al. 2005 | ★ | 0 | 0 | ★ | ★ | 0 | ★ | 0 | ★ | **5★** |
| de Leeuw JW et al. 2007 | ★ | ★ | ★ | ★ | ★ | ★ | ★ | 0 | 0 | **7★** |
| Macleod M et al. 2008 | ★ | ★ | ★ | ★ | ★ | ★ | ★ | 0 | ★ | **8★** |
| Murphy DJ et al. 2008 | ★ | ★ | 0 | ★ | ★ | 0 | ★ | 0 | 0 | **5★** |
| Revicky V et al . 2010 | ★ | ★ | ★ | ★ | ★ | ★ | ★ | 0 | 0 | **7★** |
| de Vogel j et al 2012 | ★ | ★ | ★ | ★ | ★ | ★ | ★ | 0 | ★ | **7★** |
| Räisänen S et al 2012 | ★ | ★ | 0 | ★ | ★ | ★ | ★ | 0 | ★ | **7★** |
| Yamasato K et al. 2016 | ★ | 0 | ★ | ★ | ★ | 0 | ★ | 0 | ★ | **6★** |
| van Bavel J et al 2017 | ★ | ★ | ★ | ★ | ★ | 0 | ★ | ★ | ★ | **8★** |
| Boujenah J et al. 2019 | ★ | ★ | ★ | ★ | ★ | 0 | ★ | 0 | ★ | **7★** |
| Gonzalez-Díaz E et al. 2019 | ★ | 0 | 0 | ★ | ★ | ★ | ★ | 0 | 0 | **5★** |
| Gacho B et al 2019 | ★ | ★ | 0 | ★ | ★ | 0 | ★ | 0 | ★ | **6★** |
| Muraca GM et al 2019 | ★ | ★ | ★ | ★ | ★ | 0 | ★ | 0 | ★ | **7★** |
| Frenette P et al 2019 | ★ | 0 | ★ | ★ | ★ | ★ | ★ | 0 | 0 | **6★** |
| Schreiber H et al 2021 | ★ | ★ | ★ | ★ | ★ | ★ | ★ | 0 | ★ | **8★** |
| Desplanches T et al 2021 | ★ | ★ | ★ | ★ | ★ | ★ | ★ | 0 | ★ | **8★** |
| Morgan R et al 2021 | ★ | 0 | 0 | ★ | ★ | 0 | ★ | 0 | ★ | 5★ |
| Perrin A et al 2022 | ★ | 0 | 0 | ★ | ★ | 0 | ★ | 0 | ★ | 5★ |
| Evangelopoulos N et al 2023 | ★ | 0 | 0 | ★ | ★ | 0 | ★ | 0 | ★ | 5★ |
| Ragusa A et al 2023 | ★ | ★ | ★ | ★ | ★ | 0 | ★ | 0 | ★ | 7★ |
| Bergendahl S et al. 2023 | ★ | ★ | ★ | ★ | ★ | ★ | ★ | ★ | 0 | 9★ |
